# Assessment of diagnostic value of preoperative elastography in thyroid nodules having indeterminate cytology results

**DOI:** 10.3906/sag-2101-246

**Published:** 2021-07-21

**Authors:** Bekir UÇAN, Mustafa ŞAHİN, Binnur ÖNAL, Muhammed KIZILGÜL, Hakan DÜĞER, Muhammed Erkam SENCAR, Erman ÇAKAL, Mustafa ÖZBEK

**Affiliations:** 1 Department of Endocrinology and Metabolism, Ankara Dışkapı Training and Research Hospital, University of Health Sciences, Ankara, Turkey; 2 Department of Endocrinology and Metabolism, Faculty of Medicine, Ankara University, Ankara, Turkey; 3 Department of Pathology & Cytology, Faculty of Medicine, Düzce University, Düzce, Turkey

**Keywords:** Elastography, indeterminate cytology, malignancy, thyroid

## Abstract

**Background/aim:**

The management of nodules with indeterminate cytology [atypia of undetermined significance (AUS), follicular lesion of undetermined significance (FLUS), follicular neoplasm (FN), suspicious for a follicular neoplasm (SFN), and suspicious for malignancy (SM)] results is controversial. To assess the role of the elastography technique in the diagnosis of malignancy in the subtypes of indeterminate thyroid nodules.

**Materials and methods:**

We included 132 patients with indeterminate cytology who underwent thyroid surgery. Sensitivity, specificity, area under the curve, and optimal cut-off points were calculated with receiver operating characteristic (ROC) analysis for elastography score (ES) and strain index (SI).

**Results:**

Malignancy was observed in 27/95 (28.4%) of the AUS-FLUS cytology and 12/24 (50%) of FN, SFN cytology. All of the 13 patients (100 %) with SM are found to be malignant on histology. In the FLUS group, nodules with ES greater or equal to 3, the presence of malignancy was higher 17/41 (41.5%) when compared with nodules with ES smaller than 39/46 (19.6 %) (p = 0.023). In the SFN group, 2 of 2 nodules with an ES score of 4 and 1 of 1 nodule with an ES score of 5 were malignant. In the FLUS group, 4 of 10 nodules with an ES score of 4 and 2 of 2 nodules with an ES score of 5 were malignant.

**Conclusion:**

Thyroid elastography may reduce unnecessary surgery for both patients with AUS/FLUS and selected SFN cytology. Elastography appears to be helpful in follicular variants and other types of papillary thyroid cancer, however, not in follicular thyroid cancer.

## 1. Introduction

Solitary thyroid nodules are seen as a common health problem although only 4% – 6.5% of them are diagnosed with cancer in the general population [1]. FNAB (Fine needle aspiration biopsy) is a safe procedure to diagnose thyroid malignancy preoperatively, practiced widely to a high standard [2–4]. Performing ultrasound-guided FNAB on solitary nodules has become an acceptable method rather than the conventional one [5]. The procedure is a useful tool even in infra-centimetric nodules [6]. Indeterminate cytology [atypia of undetermined significance (AUS), follicular lesion of undetermined significance (FLUS), follicular neoplasm (FN), suspicious for a follicular neoplasm (SFN), and suspicious for malignancy (SM)] accounts for approximately 20% to 25% of thyroid aspirates are found to be indeterminate results [7]. Although the aforementioned patients undergo surgery, most of them are still found to be benign [8,9]. The prevalence of malignancy in these patients varies from 15% to 51.7% according to the presence of atypia [10]. Therefore, the indeterminate diagnostic category has been a major challenge for both pathologists and clinicians [11].

Adjunctive methods, for example, molecular analysis and elastography technique are used to increase diagnostic yield [12–17]. In addition to sonographic and cytological features, elastography may increase the sensitivity for predicting malignancy [18,19].

Elastography is a useful method used to measure the stiffness of thyroid nodule compared to adjacent thyroid tissue. Malignant nodules are stiffer than benign nodules [15–18]. Elastography score (ES) and strain index (SI) is seen to be higher in malignant nodules [17,20]. In a recent study, the risk of malignancy increased approximately 2-fold for every unit increase in SR [21]. The reports about the usefulness of elastography in patients with indeterminate cytology are controversial [15–17,22].

The assessment of detailed cytological findings of the above-mentioned ‘follicular-patterned thyroid lesions’ has not been sufficient for differential diagnosis [23]. Besides the fact that sonographic features are generally not so helpful while differentiating between follicular adenoma and carcinoma, elastography may be more helpful in this subgroup.

In this study, we aimed to evaluate the diagnostic value of elastography to predict malignancy in thyroid nodules with indeterminate cytology. We also aimed to evaluate whether there is any cancer risk using histopathology as the gold standard in these subtypes or not.

## 2. Materials and methods

In our prospective study, we included 132 nodular goiter patients (13 males and 119 females) with a mean age of 44 ± 11.7 years. They all underwent surgery due to indeterminate cytology following FNAB at the Endocrinology outpatient clinic of Ankara Dışkapı Training & Research Hospital. The study received approval from the local ethical committee of Ankara Dışkapı Training & Research Hospital. Following the approval of the ethical committee, written informed consent was obtained from each patient.

Thyroid ultrasound and elastographic examinations were performed before FNAB using an EUB-7000HV scanner (Hitachi Medical Corporation, Tokyo, Japan) with a 6- to 13-MHz linear array transducer. Power Doppler examination was also performed using the same technology.

We evaluated the echogenicity, size, volume, margin regularity, presence and nature of halo, calcifications, the blood flow pattern of all nodules. Blood flow patterns were classified as type 1 if blood flow is absent, type 2 if there is only perinodular blood flow, type 3 if there is marked intranodular blood flow with absent or slight perinodular flow [24]. Itoh’s elasticity score scale was used for the measurement of ES according to different nodule color patterns [25].

We selected an area manually along the borderline of the nodule then we selected a similar-sized area beside the nodule in thyroid tissue as a reference. The software calculated the strain index automatically. We took the average of three consecutive measurements. All measurements were made by three experienced endocrinologists in this respect. The longitudinal view was used for measurement.

FNAB was performed under ultrasound assistance using a 25–27-gauge needle attached to a 10 mL syringe. On average, 2–4 interventions have been made for each nodule and all aspirates were directly smeared on glass slides for cytomorphological examination. Then the prepared slides were air-dried to be stained with May-Grunwald-Giemsa or alcohol fixed to be stained with Papanicolaou & Hematoxylin-Eosin.

Following thyroidectomy, thyroid tissue was immediately fixed in 10% buffered formalin to be processed and embedded in paraffin to prepare 5 μ-sections to be stained by Hematoxylin-Eosin. All cytological and histopathological evaluations were performed by the expert (cyto)pathologist, blinded to the elastography results. Reporting Thyroid Cytopathology 2017 was used for the cytological diagnoses and widely recognized guidelines were used for the histopathological diagnoses [9].

The G-Power 3.1 software program was used to calculate the sample size. The α level, power, and effect size were set as 0.05, 0.95, and 0.4, respectively, which indicated a minimum sample size of 134.

All statistical analysis was performed using SPSS Statistics (IBM Corporation, Somers, NY) software, version 20.0. The normality of the distribution of continuous variables was determined using the Kolmogorov–Smirnov test. The t-test was used for normally distributed continuous variables, and the Mann–Whitney U test was used for those that did not fit a normal distribution. Chi-square test or Fisher exact test were used when the variables were categorical. Logistic regression analysis was also performed to evaluate the relationship between the above parameters. The sensitivity and specificity values of elastography (score and strain index) for diagnosing malignancy were calculated and the best cut of values for them was calculated by receiver operating characteristic (ROC) analysis. The area under the ROC curves for elastography scores (ES) and strain index (SI) was calculated. Statistical significances were considered if p values were < 0.05.

## 3. Results

The mean age of patients was 44 ± 11.7 years, mean TSH was 1.96 (min 0.01-max 16) IU/mL, mean free T4 1.04 (0.55–3.72) ng/dL. The total mean thyroid volume was 20.01 ml (4.7–77.8), mean nodule volume was 5.71 (0.05–70) mL. The mean maximum diameter of the tumor on histopathology was 16.6 mm (0.3–55 mm). Malignancy was observed in 27/95 (28.4%) of the patients with atypia with undetermined significance and follicular lesion of undetermined significance (AUS-FLUS) cytology and 12/24 (50%) of patients with follicular neoplasm (FN) and suspicious for a follicular neoplasm (SFN) cytology. All of the 13 patients (100 %) with suspicious of malignancy (SM) are found to be malignant on surgical histopathology examination (Table 1). Two of the 27 patients with the AUS-FLUS category are reported as follicular thyroid cancer (FTC); 25/27 of them were reported as papillary thyroid carcinoma (PTC). 7/12 of SFN are diagnosed with PTC. 3 of them were diagnosed with FTC, and 1 of them was diagnosed with hurtle cell carcinoma. All nodules with suspicious of malignancy were diagnosed with PTC.

Elastography scores and logarithmic-strain index are found to be statistically different according to histopathology results. In the whole group, logarithmic SI was greater in patients with malignancy (p < 0.0001). Similarly, the malignancy rate was higher in the patients with ES scores greater than 4 (p = 0.001; Fischer’s exact test). Other ultrasonographic characteristics, including thyroid volume, nodule volume, sex, smoking, and other parameters, did not affect final histopathology results. Clinical, laboratory, and sonographic features (microcalcification, nodule echogenicity, the presence of halo, blurred margins, intranodular vascularity) that predict malignancy were not different between subgroups (Table 2 and 3).

In the AUS-FLUS group; the malignancy rate was 41.5% (17/41) among the nodules with ES greater than 3 and was 19.6% (9/46) among nodules with ES score smaller than 3 (p = 0.023; Table 4). Malignancy rate was not significantly different between nodules with ES score greater than 3 or nodules with ES score smaller than 3 in the SFN group (Table 4). In the SFN group, 2 nodules with an ES score of 4 and 1 nodule with an ES score of 5 were found to be malignant. In the FLUS group, 4 of 10 nodules with an ES score of 4 and 2 of 2 nodules with an ES score of 5 were found to be malignant. The specificity of elastography scoring (greater or equal to 4) in AUS-FLUS and FN-SFN were 90% and 100%, respectively (Table 5). Only one of the FTC had an ES score greater than 4.

**Figure 1:**
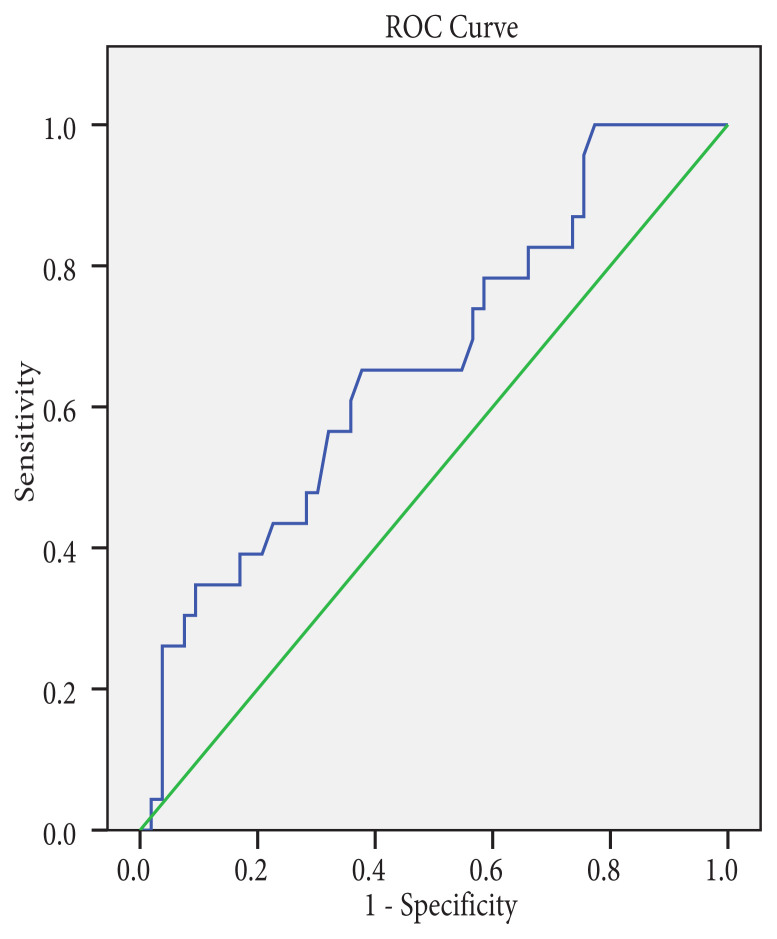
Receiver operating characteristic curve (ROC) of strain index for AUS-FLUS to distinguishing malignant from benign nodules. The area under the curve for diagnosing malignancy was 0.662 (95% confidence interval 52.9–79.5; p < 0.026).

**Figure 2:**
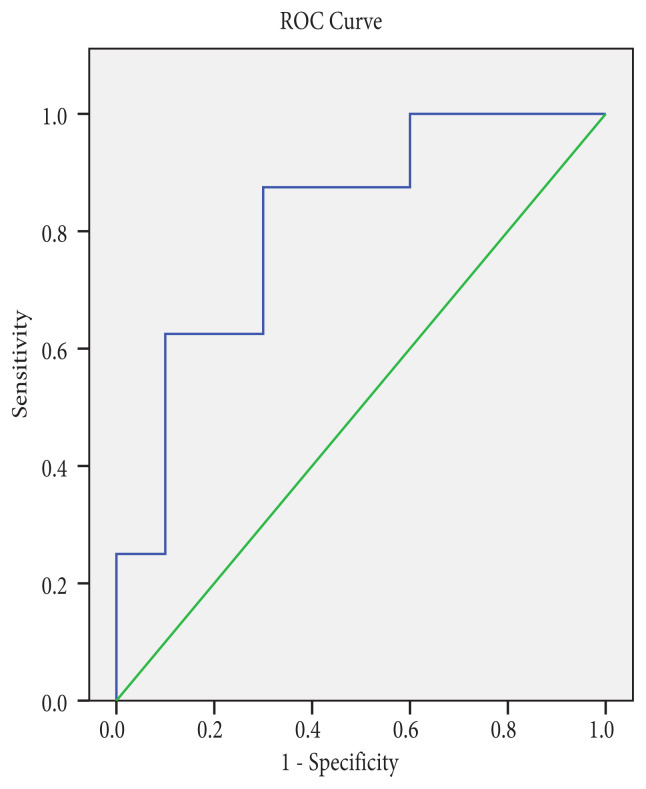
Receiver operating characteristic curve (ROC) of strain index for FN-SFN to distinguishing malignant from benign nodules. The area under the curve for diagnosing malignancy was 0.813 (95 % confidence interval 0.610–1.015, *p <* 0.0026).

**Figure 3:**
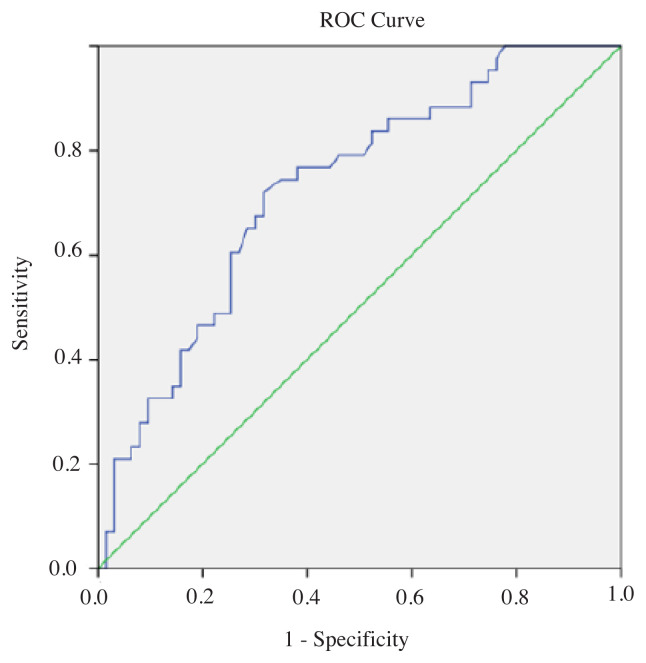
Receiver operating characteristic curve (ROC) of strain index for the whole indeterminate group to distinguishing malignant from benign nodules. The area under the curve for diagnosing malignancy was 0.727 (95 % confidence interval 0.631–0.823, p < 0.0001).

The area under the ROC curve for diagnosis of malignancy by measuring the SI value of nodules with FLUS cytology was 0.66 (95% confidence interval 52.9–79.5) (Figure 1). The area under the curve of SI for SFN and the whole group to differentiate malignant from benign nodules were 0.813 (95 % confidence interval 0.610–1.015, p < 0.0026) and 0.727 (95 % confidence interval 0.631–0.823, p < 0.0001), respectively (Figure 2, 3). Table 6 shows the best cut-off points and corresponding sensitivity, specificity values of SIs for diagnosis of malignancy. The cut-off point of 2.14 for SI in the FLUS group had a sensitivity of 65.2% and specificity of 62.3 % and 2.17 for SI in the FN-SFN group had a sensitivity of 62.5% and specificity of 90%.

## 4. Discussion

In this study, we found a high prevalence of cancer in patients with AUS-FLUS (28.4%) and FN-SFN (50%) cytology results. Currently, there is no specific tool for predicting malignancy rate in nodules with indeterminate cytology. Studies performed in nodules with indeterminate cytology showed that suspicious features in ultrasonography guide the decision for surgery.

Our study shows that ES score and SI measurements are good predictors of malignancy. In contrast to previous studies [16–18,26], clinical, laboratory, and sonographic features (microcalcification, nodule echogenicity, the presence of halo, blurred margins, intranodular vascularity) that predict malignancy were not different between subgroups of patients with indeterminate cytology.

To the best of our knowledge, our study is one of the few studies in the literature evaluating the diagnostic value of elastography specifically in patients with the AUS-FLUS cytology. According to our study, elastography is a useful diagnostic tool in patients with SFN cytology despite the previous reports with conflicting results. Additionally, the sensitivity of elastography scores for the diagnosis of malignancy in patients with SFN and AUS-FLUS cytology was low. Using an ES score greater than 4 for the diagnosis of malignancy may increase the specificity. SI had greater sensitivity for indeterminate nodules but not as high as previously reported studies. We determined a cut-off value of 2.14 for AUS-FLUS and 2.17 for SFN based on the receiver operator characteristics (ROC) analysis to differentiate malignancy. The sensitivity of these cut-off SI values was similar in both groups, but specificity was higher in the SFN group. These cut-off values were lower than the cut-offs values reported in previously published studies [27].

In our study, ES scores in patients with follicular thyroid carcinoma (FTC) were low. ES score of 4 was found in only one case. Besides, follicular thyroid carcinoma generally lacks specific features of malignancy in conventional ultrasonography and molecular analysis may be helpful in these patients. Generally, elastography seems to be more helpful in follicular type papillary thyroid cancers and all other papillary thyroid cancers. Many of the patients in the SFN group have been diagnosed with follicular type papillary thyroid carcinoma instead of FTC.

Although the Bethesda System for Reporting Thyroid Cytopathology’ 2017 provides a better-defined cancer risk in cytologically indeterminate nodules, it may not reliably predict the malignant or benign result in final histopathology. Since the lack of a definitive diagnosis for these nodules, most patients with indeterminate cytology undergo diagnostic surgery to establish the histopathological diagnosis. However, only 10%–40% of surgically resected thyroid nodules are proved to be malignant [17]. The diagnostic operations, with their attendant expenses and risks, could be avoided if the presurgical diagnosis of these indeterminate nodules could reliably exclude the malignancy.

Lobectomy may not be suitable for indeterminate nodules in our population because many of them were diagnosed with PTC rather than follicular tumors.

Limitations: Since the number of our SFN cases was low for statistical evaluation, prospective studies including more patients with SFN cytology results are needed.

The elastography technique is valuable in the diagnosis of malignancy in a thyroid nodule with indeterminate cytology especially if the lesion lacks the malignant features in conventional ultrasonography. However, a low ES score does not rule out malignancy risk in these nodules. More powerful predictors of malignancy in these subgroups (SFN and AUS-FLUS) of indeterminate nodules are still needed.

## Figures and Tables

**Table 1:** Distribution of carcinoma rate in final histopathological diagnosis according to subtype of indeterminate FNAB cytology.

	AUS-FLUS	FN-SFN	SM
Carcinoma rate	27/95 (%28.4)	12/24 (%50)	13/13 (%100)
Papillary TCA	25	7	13
FTC/HTC	2	5	

**Abbreviations:** Atypia of unknown significance (AUS), Atypia with undetermined significance (FLUS), Follicular neoplasia (FN) and suspicious of follicular neoplasm cytology (SFN), suspicious for malignancy (SM).

**Table 2:** Demographic, clinical, and other laboratory characteristics according to malignancy in final histopathological diagnosis in AUS-FLUS subtype of indeterminate FNAB cytology.

	Benign	Malign	*p*
**Age**	44.27 ± 12.1	46.5 ± 11.5	*
**Gender**	8M/60F	2M/25F	*
**TSH**	2.1 (1.5–2.8)	1.8 (1.07–2.5)	*
**Free T4**	1.06 (0.9–1.17)	0.98 (0.92–1.03)	*
**Log-anti-TPO**	1.87 ± 0.8	1.5 ± 0.6	*
**Log anti-Tg**	1.52 ± 0.7	1.73 ± 0.6	*
**Log-Total volume**	1.33 ± 0.27	1.36 ± 0.27	*
**Log-Nodule volume**	0.33 (0.17–0.49)	0.39 (0.06–0.7)	*
**Log-SI**	0.28 (0.19–0.36)	0.45 (0.32–0.57)	**0.023**

* p>0.05 Strain index (SI) = strain of thyroid tissue / strain of nodular lesion

**Table 3:** Demographic, clinical, and other laboratory characteristics according to malignancy in final histopathological diagnosis in FN-SFN subtype of indeterminate FNAB cytology.

	Benign	Malign	*p*
**Age**	44.27 ± 12.1	46.5 ± 11.5	*
**Gender**	8M/60F	2M/25F	*
**TSH**	2.1 (1.5–2.8)	1.8 (1.07–2.5)	*
**Free T4**	1.06 (0.9–1.17)	0.98 (0.92–1.03)	*
**Log-anti-TPO**	1.87 ± 0.8	1.5 ± 0.6	*
**Log anti-Tg**	1.52 ± 0.7	1.73 ± 0.6	*
**Log-Total volume**	1.33 ± 0.27	1.36 ± 0.27	*
**Log- Nodule volume**	0.33 (0.17–0.49)	0.39 (0.06–0.7)	*
**Log-SI**	0.28 (0.19–0.36)	0.45 (0.32–0.57)	**0.023**

* p>0.05

**Table 4:** Distribution of elastography scores according to fine needle aspiration cytology results and final histopathological diagnosis.

	ES score	Benign	Malign	p
AUS-FLUS	ES <3	37 (%80.4)	9 (%19.6)	**0.023**
	ES≥3	24 (%58.5)	17 (%41.5)	
FN-SFN	ES <3	9 (%69.3)	4 (%30.7)	0.063
	ES≥3	2 (%25)	6 (%75)	
SM	ES≥3	0	12 (%100)	

**Table 5:** Sensitivity and specificity of elastography scoring (greater or equal to 4) in indeterminate nodules.

	Sensitivity (%)	Specificity (%)
Whole group	33.3	91.55
AUS-FLUS	23.0	90
FN-SFN	30	100

**Table 6:** Sensitivity and specificity of elastosonography index.

	SI best cut-off	Sensitivity (%)	Specificity (%)
AUS-FLUS	2.14	65.2	62.3
FN-SFN	2.17	62.5	90
Whole	2.48	60.5	74.6
